# Electronic Structures of Silicene Nanoribbons: Two-Edge-Chemistry Modification and First-Principles Study

**DOI:** 10.1186/s11671-016-1584-5

**Published:** 2016-08-22

**Authors:** Yin Yao, Anping Liu, Jianhui Bai, Xuanmei Zhang, Rui Wang

**Affiliations:** 1 Department of Physics and Institute for Structure and Function, Chongqing University, Chongqing, People’s Republic of China; 2 Department of Environmental Physics, Chongqing University, Chongqing, 24105 People’s Republic of China

**Keywords:** Silicene, Electronic structure, Localized edge states, Density functional theory

## Abstract

In this paper, we investigate the structural and electronic properties of zigzag silicene nanoribbons (ZSiNRs) with edge-chemistry modified by H, F, OH, and O, using the ab initio density functional theory method and local spin-density approximation. Three kinds of spin polarized configurations are considered: nonspin polarization (NM), ferromagnetic spin coupling for all electrons (FM), ferromagnetic ordering along each edge, and antiparallel spin orientation between the two edges (AFM). The H, F, and OH groups modified 8-ZSiNRs have the AFM ground state. The directly edge oxidized (O_1_) ZSiNRs yield the same energy and band structure for NM, FM, and AFM configurations, owning to the same *sp*^2^ hybridization. And replacing the Si atoms on the two edges with O atoms (O_2_) yields FM ground state. The edge-chemistry-modified ZSiNRs all exhibit metallic band structures. And the modifications introduce special edge state strongly localized at the Si atoms in the edge, except for the O_1_ form. The modification of the zigzag edges of silicene nanoribbons is a key issue to apply the silicene into the field effect transistors (FETs) and gives more necessity to better understand the experimental findings.

## Review

### Introduction

As the silicon counterpart of graphene [1], silicene [2–5] becomes a hot spot in low-dimension material application after being recently grown on different metallic surfaces [6–12], which consists of a single layer of Si atoms arranged in a hexagonal network as well as low-buckled geometry. The buckled distance is 0.4 Å between the layers, and this low-buckled structure is attributed to the pseudo-Jahn-Teller distortion [13–15]. Theoretical calculations have revealed that *sp*^3^ hybridization and *sp*^2^ hybridization in a low-buckled silicene structure is different from the *sp*^2^ hybridization of planer geometry in graphene [9, 16–18]. Tao et al. have successfully fabricated the first silicene-based field-effect transistors (FETs) operating at room temperature [19] and make a breakthrough for applications of silicene.

Because of its extraordinary electronic properties, silicene nanoribbons (SiNRs)[20–22] also attracts lots of attentions. The electronic properties of silicene nanoribbons are dependent on the structural size and chirality. Via assistances of recent investigations [23–26], the ZSiNRs exhibit rich electronic transport, magnetic properties, and may be applied in spintronic nanodevices potentially [27–32]. In particular, electronic transport properties of the SiNRs are vital in electronic industry [33–36]. Zigzag edges have a localized edge state at the Fermi level with semimetal properties [35–37]. Therefore, modifications of the ZSiNRs with chemical elements is important. Silicon atoms in silicene tend to adopt *sp*^3^ hybridization over *sp*^2^, which makes it extremely reactive towards O_2_ and H _2_O and also has a tendency to self-aggregate. This can be suitably be avoided by using Ca intercalation [38]. The effect of zigzag edges chemistry of graphene has been studied with an edge-modified form C–H (*sp*^2^*σ*-bonded edge C) and represents ferromagnetic edge magnetization [39]. The similar significant properties may exist in the edge-chemistry-modified silicene nanoribbons, so we choose H, F, OH, and O atoms or atomic groups for investigation.

In this paper, we report the simulations of the geometry structures and electronic properties of the 8-ZSiNRs (with eight zigzag armchair chains) with edge-chemistry modifications by H, F, OH, and O atoms or atomic groups. We calculate three kinds of spin-polarized SiNRs: nonspin polarization (NM), ferromagnetic spin coupling for all electrons (FM), and anti-parallel spin orientation between the two edges (AFM). The electronic band structures of ZSiNRs with H, F, and OH edges have similar profile due to their same *sp*^2^ hybridization. The band gap opening of these edge modifications are no larger than 0.14 eV, which is very small, indicating the exhibition of metallic properties. Except the directly oxidized (O_1_) ZSiNRs, other modifications all have special edge state strongly localized at the Si atoms in the edge. The H, F, and OH groups’ modified 8-ZSiNRs have the AFM ground state. The directly edge oxidized (O_1_) ZSiNRs yield the same energy and band structure for NM, FM, and AFM configurations. And replacing the Si atoms on the two edges with O atoms (O_2_) yield FM ground state. The modification of the zigzag edges of silicene nanoribbons is a key issue in applying the silicene into the FETs and gives more necessary to better understand the experimental findings.

### Method

The electronic properties are studied through the DFT calculations with the local density approximation (LDA) [40] and the projector-augmented wave (PAW) [41, 42] potentials, implemented as the Vienna ab initio simulation package (VASP) [43, 44]. A plane-wave basis set with kinetic energy cutoff of 400 eV has been used. In the structure optimizations and relaxations, a mesh of 13×1×1 Monkhorst-Pack [45] special *k* points is chosen for the Brillouin zone integrations. The vacuum separation between the nanoribbons in the adjacent unit cells is chosen to be 15 Å. All positions of the atoms and the lattice constant are optimized by minimization of the total energy and atomic forces. The convergence for energy is chosen as 10^−6^ eV between two steps. For the density of states (DOS) calculations, we used the tetrahedron integration method with a Monkhorst pack special 19×1×1*k* grid followed by the broadening with the Gaussian width of 0.05 eV. All of the ZSiNRs are studied with the width of eight zigzag chains (*N*_*Z*_=8,*L*=2.97 nm) with 16 Si atoms for the bare part. We consider four kinds of chemical edge modification (H, F, OH, O). Three kinds of spin-polarized calculation are performed for all of the ZSiNRs: NM, FM, and AFM.

### Results

**Fig. 1:**
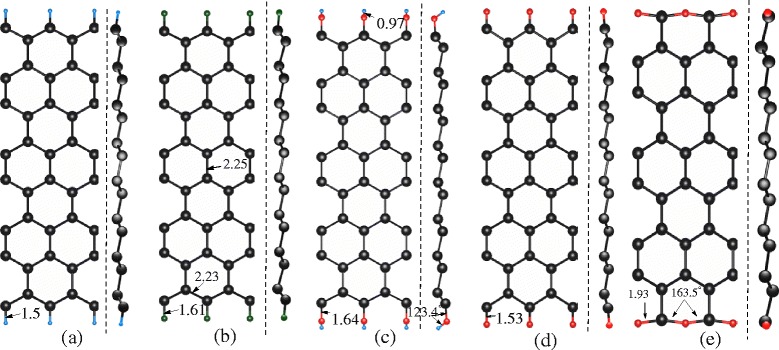
Structures. The *top* and *side* views of the no spin polarization (NM) ZSiNRs with edge-chemistry modification by **a** H, **b** F, **c** OH, **d** O_1_: direct oxidation, and **e** O_2_: replacing two-edge Si atoms with O atoms. Some notable bond length (in angstroms) and angles (in degrees) are marked in the figure to describe the structure change of ZSiNRs with these modification. The *blue dots* represent the H atoms, the *green dots* represent the F atoms, the *red dots* represent the O atoms, and the *black dots* represent the Si atoms

In this section,we present the geometric structures and the band structures of ZSiNRs with chemical-edge modification including hydrogenation, fluorination, hydroxylation, and oxidation at both edges. The top and side views of differently modified ZSiNRs, without spin polarization, are shown in Fig. 1. The geometric structures of the hydrogenated, fluorinated, and hydroxylated ZSiNRs are similar, and each of them only has one form. The edge could be oxidized in two forms. One is the direct oxidation (referred to as O_1_). The other is a replacement of Si with O at both edges (referred to as O_2_). The energy of the edge-chemistry-modified ZSiNRs is lower than the bare ZSiNRs. In order to investigate the magnetic properties, the relative energy *E*_FM(AFM)_−*E*_NM_ of FM (AFM) configurations to the NM configurations is calculated in Table 1. The three kinds of spin-polarized configurations exhibit comparable stability as the energy difference between them is no larger than 13.17 meV. All the ZSiNRs with modification of H, F, and OH favor AFM ground states. In contrast, the ribbon with O_2_ modification prefers FM ground state. The FM and AFM configurations have nearly the same structures with the NM ones.

**Table 1:** Energy of spin-polarized configurations. The relative energy (in unit of meV) of ferromagnetic spin coupling for all electrons (FM) and anti-parallel spin orientation between the two edges (AFM) configurations to the no spin polarization (NM) configurations

Relative energy (meV)	H	F	OH	O_1_	O_2_
*E*_FM_−*E*_NM_	–7.51	–6.91	–9.06	0	–13.17
*E*_AFM_−*E*_NM_	–8.08	–7.40	–9.70	0	–0.07

#### Two-Edge Hydrogenation

**Fig. 2:**
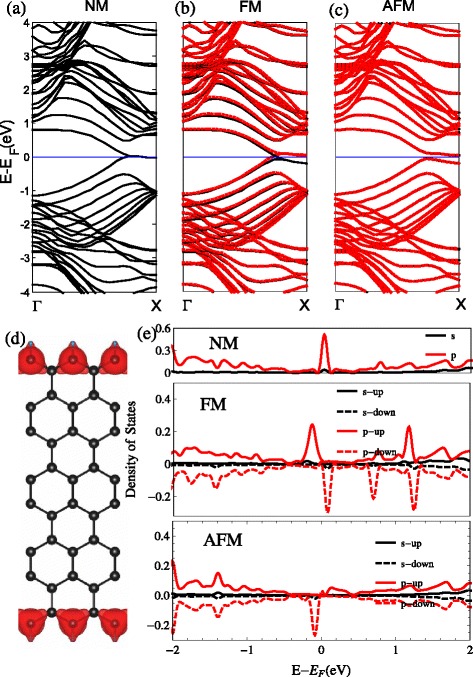
H. The band structures with the two-edge-chemistry modified by hydrogen for three kinds of spin configuration: **a** no spin polarization (NM), **b** ferromagnetic spin coupling for all electrons (FM), and **c** ferromagnetic ordering along each edge and antiparallel spin orientation between the two edges (AFM). **d** Band-decomposed charge densities of the edge states are also given (the isosurface is set to $0.0027a_{0}^{-3}$)(*a*_0_: Bohr radius). For the FM and AFM, spin-up and spin-down states are represented by the *black and red lines* in the band structure. The projected density of states (pDOS) of the Si atoms at the edge are shown in **e**

For a bare ZSiNR, the inner silicon atoms have three nearest neighbors. The Si atoms at the edge, on the other hand, have only two neighbors. As a result, the edge atoms have one dangling orbital which does not participate in the *σ* bonding. Hydrogen (H)-edge modification causes *sp*^2^- *σ* hybridized bonding. In Fig. 1a, the structure of ZSiNRs with the two-edge-chemistry modified by hydrogen (SiH–SiH) is shown in the top and side views. The length of the Si–H bonds is 1.61 Å, and the bond length between the Si atoms next to the edge is shortened to 2.23 Å. The band structures of the three types of spin-polarized configurations are plotted in Fig. 2. For the NM band structures, as we can see in Fig. 2a, the highest *π*(*π*-top) band and the lowest *π*^∗^(*π*^∗^-bottom) band degenerate at the Fermi level and have a flat region from *k*=2/3*π* to *k*=*π*, as the period of ZSiNRs is taken as *a*=1. It is well known that the flat band represents edge state, as shown in Fig. 2d, that is, the strongly localized charge density of the decomposed *π*-top band. In the NM-projected density of states (pDOS) in Fig. 2e, the *p*-orbital of the edge Si atoms shows a large peak at the Fermi level as well, which makes the main contribution. For the FM band structures, the spin-up and spin-down bands are plotted with black solid and red dashed lines, respectively, in Fig. 2b. The spin-up-degenerated bands crossing below the Fermi level near *k*=2/3*π* are occupied, and the spin-downs above the Fermi level are empty. This results in a net magnetic moment of 0.58 *μ*_*B*_. These band structure are similar to the results of Xu et al. obtained for the H-passivated ZSiNR [35], which can confirm our results and prove the correctness of our calculation. As shown in Fig. 2c, the *π*^∗^-bottom and *π*-top bands move up and down, respectively, for the AFM band structures. The spin-up and spin-down bands still keep degeneracy, which means zero total magnetism. But the AFM pDOS of edge Si atoms in Fig. 2e shows ferromagnetic ordering in each edge. Although there is a small indirect band gap opening of 0.11 eV, the AFM ZSiNRs exhibit metallic properties as the NM and FM configurations as well.

#### Edge Fluoridation and Hydroxylation

**Fig. 3:**
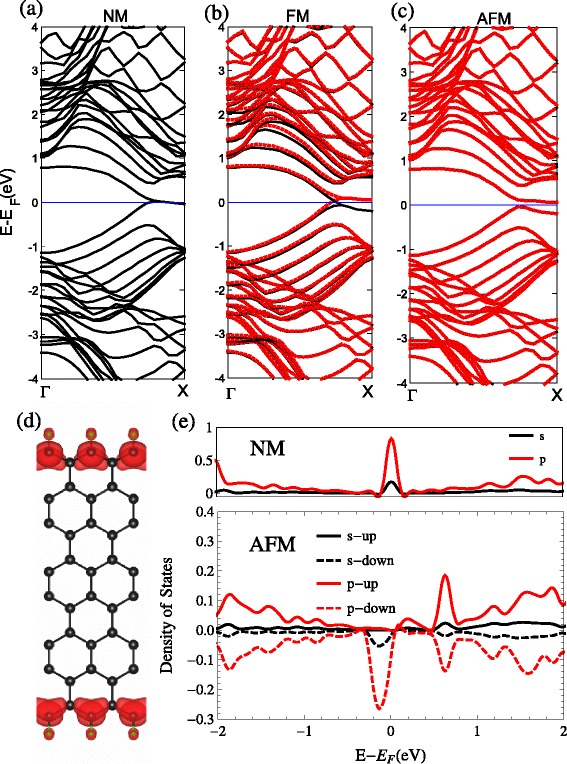
F. The band structures with the two-edge-chemistry modified by fluorine for three kinds of spin configuration: **a** no spin polarization (NM), **b** ferromagnetic spin coupling for all electrons (FM), and **c** ferromagnetic ordering along each edge and antiparallel spin orientation between the two edges (AFM). **d** Band-decomposed charge densities of the edge states are also given (the isosurface is set to $0.0027a_{0}^{-3}$)(*a*_0_: Bohr radius). For the FM and AFM, spin-up and spin-down states are represented by the *black and red lines* in the band structure. The projected density of states (pDOS) of the Si atoms at the edge are shown in **e**

**Fig. 4:**
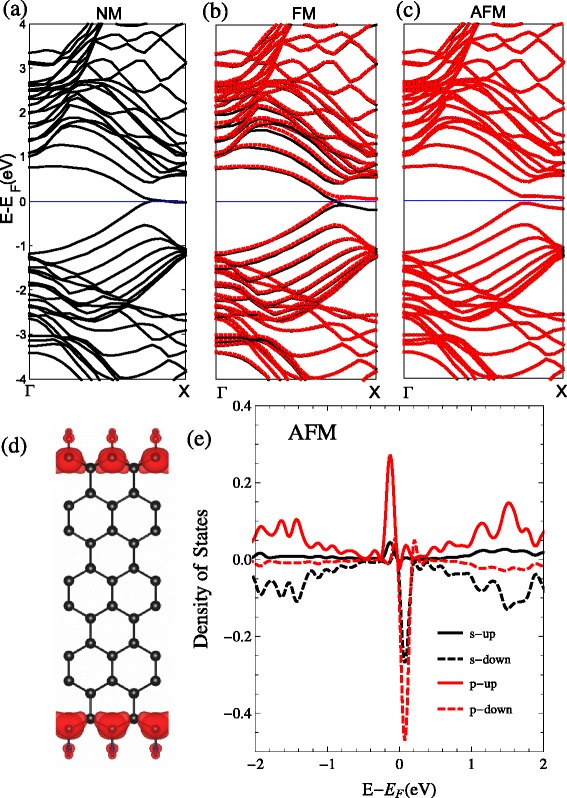
OH. The band structures with the two-edge-chemistry modified by hydroxyl for three kinds of spin configuration: **a** no spin polarization (NM), **b** ferromagnetic spin coupling for all electrons (FM), and **c** ferromagnetic ordering along each edge and antiparallel spin orientation between the two edges (AFM). **d** Band-decomposed charge densities of the edge states are also given (the isosurface is set to $0.0027a_{0}^{-3}$)(*a*_0_: Bohr radius). For the FM and AFM, spin-up and spin-down states are represented by the *black and red lines* in the band structure. The projected density of states (pDOS) of the Si atoms at the edge are shown in **e**

The ZSiNRs with edge oxidation by fluorine (F) and hydroxyl (OH) have similar electronic properties with hydrogen-modified ZSiNRs. This results from the similar *sp*^2^- *σ* hybridized bonding in the edge; see Figs. 3 and 4. For instance, the NM band structures have obvious edge states, there is degeneracy breaking between the two spins for the FM configurations and the indirect band gap opening for the AFM configurations. The net magnetic moment of FM configurations are 0.56 *μ*_*B*_ for fluoridation and 0.59 *μ*_*B*_ for hydroxylation. The indirect band gap of the AFM configurations are 0.08 eV for fluoridation and 0.14 eV for hydroxylation. The other difference of band structures in Figs. 2, 3 and 4 can be barely seen.

#### Edge Oxidation

The ZSiNRs modified with oxygen (O) atoms have two kinds of structures. One includes oxidation directly, which can be called as O_1_ (SiO–SiO). And the other has the structure with the O atoms replacing the Si atom in the two edges and is referred to as O_2_ (Si _2_O–Si _2_O). The geometric constructions are clearly shown in Fig. 1d, e. Notably, replacing the Si atoms on the edge with O atoms can be one method to change the buckling structure of silicene.

##### Oxidation Directly (O_1_)

**Fig. 5:**
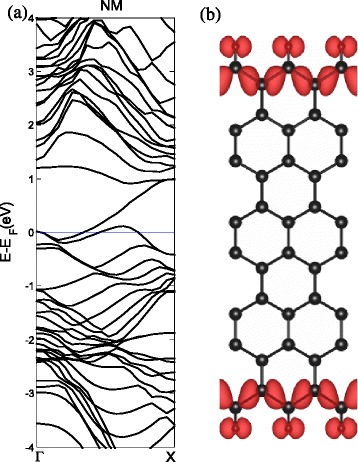
O_1_. **a** The non-spin-polarized band structures with the two-edge-chemistry modified by oxidation directly (O_1_: Si–O), which is almost the same with that of FM and AFM configurations. **b** Band-decomposed charge densities of the edge states (the isosurface is set to $0.0027a_{0}^{-3}$)(*a*_0_: Bohr radius)

The band structure of the non-spin polarized configuration is shown in Fig. 5a. Surprisingly, the NM, FM, and AFM band structures have almost the same profile. The two singly occupied 2*p* orbits of the O atoms are expected to form one *σ* and one *π* bond with the edge Si atoms. Actually, the *π* orbits will be passivated. Not only the band structures but also the total energies for the three spin-polarized configurations are the same. Their spin magnetic moments are nearly zero. The highest *π*(*π*-top) bands and the lowest *π*^∗^(*π*^∗^-bottom) bands are degenerated at the *Γ* point with zero band gap. They exhibit metallic properties because the *π*-top band has been half filled and the *π*^∗^-bottom band is below the Fermi level. From Fig. 5b, the electrons are not that highly localized at the Si atoms in the edges.

##### Replacing the Si Atom with O Atom (O_2_)

**Fig. 6:**
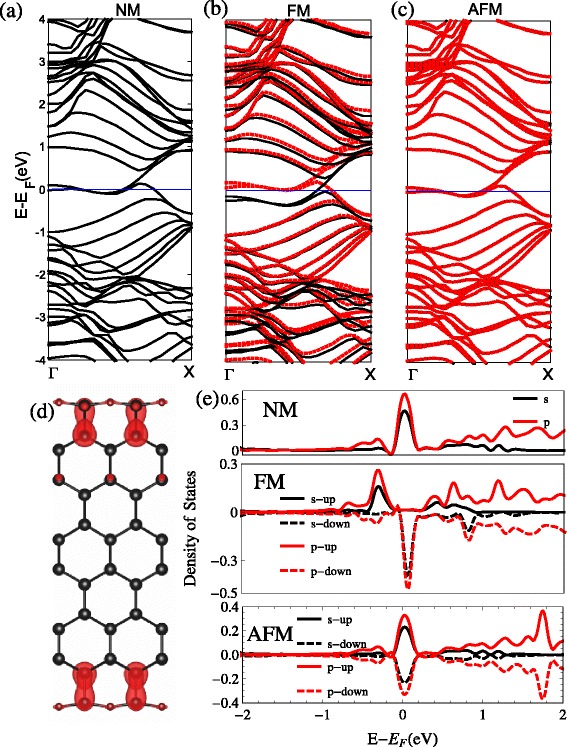
O_2_. The band structures with the two-edge-chemistry modified by the oxygen atoms replaced the Si atoms (O_2_: Si–O–Si) for three kinds of spin configuration: **a** no spin polarization (NM), **b** ferromagnetic spin coupling for all electrons (FM), and **c** ferromagnetic ordering along each edge and antiparallel spin orientation between the two edges (AFM). **d** Band-decomposed charge densities of the edge states are also given (the isosurface is set to $0.0027a_{0}^{-3}$)(*a*_0_: Bohr radius). For the FM and AFM, spin-up and spin-down states are represented by the *black and red lines* in the band structure. The projected density of states (pDOS) of the Si atoms at the edge are shown in **e**

It shows the geometric construction of the ZSiNRs by replacing the Si atoms with O atoms in each edge in Fig. 1e. The result of the band structures with the three types of spin polarizations is shown in Fig. 6. It is clear that the two degenerate bands across the Fermi level near *k*=0.2*π* and *k*=0.5*π* with zero band gap for the NM band structures. For the FM band structures, the spin-up and spin-down bands move down and up, respectively, which results in a much larger net magnetic moment of 1.34 *μ*_*B*_. There is a small band gap opening about 0.025 eV of the AFM band structure. This results in a net spin magnetic moment of zero. The total energies for the FM and AFM configurations are lower than that of NM configuration by 13.17 and 0.07 meV. From the *π*-top band-decomposed charge density in Fig. 6d and the peak of the pDOS of edge atoms in the Fermi level in Fig. 6e, the *p*-orbital and *s*-orbital both make contribution for the highly localized *π* bands at the atoms in edges. Due to the different bonding between the O_1_ and O_2_ forms, they have different band structures, and there may be Si =O double bonds in the O_1_ configuration and Si–O single bond in the O_2_ configuration.

## Conclusions

We have calculated the band structures and the pDOS of the ZSiNRs with the two-edge-chemistry modified by the various functional groups: H, F, OH, and O. Three types of spin-polarized configurations (NM, FM, AFM) are considered. It is found that the electronic band structures of ZSiNRs oxidized by the F and OH groups have very similar profile with that of H passivation, due to the same *sp*^2^ hybridization for each edge Si atom. It also results in the same effect of spin polarization, that is, the H, F, and OH groups’ modified ZSiNRs all have the AFM ground state. There are special edge states that strongly localized at the Si atoms in the edge, which especially originates from their *p* orbital electrons. The ZSiNR edge modified by O atoms directly (SiO–SiO) yields the same band structure for the three types of spin polarization (NM, FM, AFM), which means it is non-magnetic. Since the different spin configurations do not shift the electronic energy level, the calculated total energies are almost the same, and their spin magnetic moments are zero. One of the two singly occupied 2*p* orbitals of the O atom forms one *σ* bond with the edge Si atom. The other one left occupies the *π*-top (*π*∗-bottom) state. For replacing the Si atoms on the two edges with O atoms (O_2_), the two singly occupied 2*p* orbitals of the O atom forms two *σ* bonds with the inner edge Si atoms, and the edge states are highly localized at the two inner edge Si atoms. This modification yields FM ground state. The ZSiNRs edge-chemistry modified by H, F, OH, and O all exhibit metallic band structure. It is quite important to control the ZSiNR edge-chemistry modification for future applications of SiNRs.

## References

[1] Novoselov KS, Geim AK, Morozov S, Jiang D, Zhang Y, Dubonos S, Grigorieva I, Firsov A (2004). Electric field effect in atomically thin carbon films. Science.

[2] Cahangirov S, Topsakal M, Aktürk E, şahin H, Ciraci S (2009). Two- and one-dimensional honeycomb structures of silicon and germanium. Phys Rev Lett.

[3] Jose D, Datta A (2012). Understanding of the buckling distortions in silicene. J Phys Chem C.

[4] Lebègue S, Eriksson O (2009). Electronic structure of two-dimensional crystals from *ab initio theory*. Phys Rev B.

[5] Houssa M (2015). The rise of silicene. NPG Asia Mater.

[6] Vogt P, De Padova P, Quaresima C, Avila J, Frantzeskakis E, Asensio MC, Resta A, Ealet B, Le Lay G (2012). Silicene: compelling experimental evidence for graphenelike two-dimensional silicon. Phys Rev Lett.

[7] Feng B, Ding Z, Meng S, Yao Y, He X, Cheng P, Chen L, Wu K (2012). Evidence of silicene in honeycomb structures of silicon on Ag(111). Nano Lett.

[8] Jamgotchian H, Colignon Y, Hamzaoui N, Ealet B, Hoarau J, Aufray B, Bibérian J (2012). Growth of silicene layers on Ag(111): unexpected effect of the substrate temperature. J Phys Condens Matter.

[9] Ezawa M (2013). Hexagonally warped Dirac cones and topological phase transition in silicene superstructure. Eur Phys J B.

[10] Fleurence A, Friedlein R, Ozaki T, Kawai H, Wang Y, Yamada-Takamura Y (2012). Experimental evidence for epitaxial silicene on diboride thin films. Phys Rev Lett.

[11] Meng L, Wang Y, Zhang L, Du S, Wu R, Li L, Zhang Y, Li G, Zhou H, Hofer WA (2013). Buckled silicene formation on Ir(111). Nano Lett.

[12] Chiappe D, Scalise E, Cinquanta E, Grazianetti C, van den Broek B, Fanciulli M, Houssa M, Molle A (2014). Two-dimensional Si nanosheets with local hexagonal structure on a MoS _2_ surface. Adv Mater.

[13] Chowdhury C, Jahiruddin S, Datta A (2016). Pseudo-Jahn–Teller distortion in two-dimensional phosphorus: origin of black and blue phases of phosphorene and band gap modulation by molecular charge transfer. J Phys Chem Lett.

[14] Mandal TK, Jose D, Nijamudheen A, Datta A (2014). Structures and electronic properties of heavier congeners of disk-like molecules:(Si, Ge) sulflower and (Si, Ge) olympicene. J Phys Chem C.

[15] Jose D, Datta A (2013). Structures and chemical properties of silicene: unlike graphene. Acc Chem Res.

[16] Ni Z, Liu Q, Tang K, Zheng J, Zhou J, Qin R, Gao Z, Yu D, Lu J (2011). Tunable bandgap in silicene and germanene. Nano Lett.

[17] Drummond N, Zolyomi V, Fal’Ko V (2012). Electrically tunable band gap in silicene. Phys Rev B.

[18] Ezawa M (2013). Photoinduced topological phase transition and a single Dirac-cone state in silicene. Phys Rev Lett.

[19] Tao L, Cinquanta E, Chiappe D, Grazianetti C, Fanciulli M, Dubey M, Molle A, Akinwande D (2015). Silicene field-effect transistors operating at room temperature. Nat Nanotechnol.

[20] Ding Y, Ni J (2009). Electronic structures of silicon nanoribbons. Appl Phys Lett.

[21] De Padova P, Quaresima C, Ottaviani C, Sheverdyaeva PM, Moras P, Carbone C, Topwal D, Olivieri B, Kara A, Oughaddou H (2010). Evidence of graphene-like electronic signature in silicene nanoribbons. Appl Phys Lett.

[22] De Padova P, Quaresima C, Olivieri B, Perfetti P, Le Lay G (2011). sp2-like hybridization of silicon valence orbitals in silicene nanoribbons. Appl Phys Lett.

[23] Le NB, Huan TD, Woods LM (2014). Tunable spin-dependent properties of zigzag silicene nanoribbons. Phys Rev Appl.

[24] Shakouri K, Simchi H, Esmaeilzadeh M, Mazidabadi H, Peeters F (2015). Tunable spin and charge transport in silicene nanoribbons. Phys Rev B.

[25] Yang X, Liu Y, Feng J, Wang X, Zhang C, Chi F (2014). Transport properties of bare and hydrogenated zigzag silicene nanoribbons: negative differential resistances and perfect spin-filtering effects. J Appl Phys.

[26] Ding Y, Wang Y (2014). Electronic structures of reconstructed zigzag silicene nanoribbons. Appl Phys Lett.

[27] Nduwimana A, Wang X-Q (2009). Energy gaps in supramolecular functionalized graphene nanoribbons. ACS Nano.

[28] Lambin P, Amara H, Ducastelle F, Henrard L (2012). Long-range interactions between substitutional nitrogen dopants in graphene: electronic properties calculations. Phys Rev B.

[29] Wong J-H, Wu B-R, Lin M-F2012. Strain effect on the electronic properties of single layer and bilayer graphene, Vol. 116.

[30] Liu C-C, Feng W, Yao Y (2011). Quantum spin hall effect in silicene and two-dimensional germanium. Phys Rev Lett.

[31] Liu C-C, Jiang H, Yao Y (2011) Low-energy effective Hamiltonian involving spin-orbit coupling in silicene and two-dimensional germanium and tin. Phys. Rev. doi:10.1103/PhysRevB.84.195430. BarXiv preprint arXiv:1108.2933.

[32] Haskins J, Sevik C, Sevincli H, Cuniberti G, Cagin T, Kınacı A (2011). Control of thermal and electronic transport in defect-engineered graphene nanoribbons. Acs Nano.

[33] Tada K, Watanabe K (2002). Ab initio study of field emission from graphitic ribbons. Phys Rev Lett.

[34] Lin C-Y, Chen S-C, Wu J-Y, Lin M-F (2012). Curvature effects on magnetoelectronic properties of nanographene ribbons. J Phys Soc Jpn.

[35] Xu C, Luo G, Liu Q, Zheng J, Zhang Z, Nagase S, Gao Z, Lu J (2012). Giant magnetoresistance in silicene nanoribbons. Nanoscale.

[36] Kang J, Wu F, Li J (2012). Symmetry-dependent transport properties and magnetoresistance in zigzag silicene nanoribbons. Appl Phys Lett.

[37] Pan L, Liu H, Tan X, Lv H, Shi J, Tang X, Zheng G (2012). Thermoelectric properties of armchair and zigzag silicene nanoribbons. Phys Chem Chem Phys.

[38] Pratik SM, Nijamudheen A, Datta A (2015). Topochemical transformations of CaX2 (X=C, Si, Ge) to form free-standing two-dimensional materials. Chem Eur J.

[39] Lee G, Cho K (2009). Electronic structures of zigzag graphene nanoribbons with edge hydrogenation and oxidation. Phys Rev B.

[40] Ceperley DM, Alder B (1980). Ground state of the electron gas by a stochastic method. Phys Rev Lett.

[41] Blöchl PE (1994). Projector augmented-wave method. Phys Rev B.

[42] Kresse G, Joubert D (1999). From ultrasoft pseudopotentials to the projector augmented-wave method. Phys Rev B.

[43] Kresse G, Hafner J (1993). *Ab initio* molecular dynamics for liquid metals. Phys Rev B.

[44] Kresse G, Furthmüller J (1996). Efficient iterative schemes for ab initio total-energy calculations using a plane-wave basis set. Phys Rev B.

[45] Monkhorst HJ, Pack JD (1976). Special points for Brillouin-zone integrations. Phys Rev B.

